# Rectus Femoris Muscle Segmentation on Ultrasound Images of Older Adults Using Automatic Segment Anything Model, nnU-Net and U-Net—A Prospective Study of Hong Kong Community Cohort

**DOI:** 10.3390/bioengineering11121291

**Published:** 2024-12-19

**Authors:** Dawei Zhang, Hongyu Kang, Yu Sun, Justina Yat Wa Liu, Ka-Shing Lee, Zhen Song, Jien Vei Khaw, Jackie Yeung, Tao Peng, Sai-kit Lam, Yongping Zheng

**Affiliations:** 1 Department of Biomedical Engineering, The Hong Kong Polytechnic University, Hong Kong SAR, China; dawei-joy.zhang@polyu.edu.hk (D.Z.); hong-yu.kang@connect.polyu.hk (H.K.); stefanie.sun@polyu.edu.hk (Y.S.); i-ka-shing.lee@polyu.edu.hk (K.-S.L.); zhen0212.song@connect.polyu.hk (Z.S.); jien-vei.khaw@polyu.edu.hk (J.V.K.); saikit.lam@polyu.edu.hk (S.-k.L.); 2 School of Nursing, The Hong Kong Polytechnic University, Hong Kong SAR, China; justina.liu@polyu.edu.hk (J.Y.W.L.); jackie.yeung@polyu.edu.hk (J.Y.); 3 School of Future Science and Engineering, Soochow University, Suzhou 215222, China; sdpengtao401@gmail.com; 4 Research Institute of Smart Ageing, The Hong Kong Polytechnic University, Hong Kong SAR, China

**Keywords:** deep learning, medical segment anything model, rectus femoris muscle, Sarcopenia Ultrasound, U-Net

## Abstract

Sarcopenia is characterized by a degeneration of muscle mass and strength that incurs impaired mobility, posing grievous impacts on the quality of life and well-being of older adults worldwide. In 2018, a new international consensus was formulated to incorporate ultrasound imaging of the rectus femoris (RF) muscle for early sarcopenia assessment. Nonetheless, current clinical RF muscle identification and delineation procedures are manual, subjective, inaccurate, and challenging. Thus, developing an effective AI-empowered RF segmentation model to streamline downstream sarcopenia assessment is highly desirable. Yet, this area of research readily goes unnoticed compared to other disciplines, and relevant research is desperately wanted, especially in comparison among traditional, classic, and cutting-edge segmentation networks. This study evaluated an emerging Automatic Segment Anything Model (AutoSAM) compared to the U-Net and nnU-Net models for RF segmentation on ultrasound images. We prospectively analyzed ultrasound images of 257 older adults (aged > 65) in a community setting from Hong Kong’s District Elderly Community Centers. Three models were developed on a training set (*n* = 219) and independently evaluated on a testing set (*n* = 38) in aspects of DICE, Intersection-over-Union, Hausdorff Distance (HD), accuracy, precision, recall, as well as stability. The results indicated that the AutoSAM achieved the best segmentation agreement in all the evaluating metrics, consistently outperforming the U-Net and nnU-Net models. The results offered an effective state-of-the-art RF muscle segmentation tool for sarcopenia assessment in the future.

## 1. Introduction

The word “Sarcopenia” originated from the Greek roots of “sex” and “penia”, which refers to “flesh” and “loss” [1]. Sarcopenia is an age-related disease that has been receiving increasing attention worldwide, and it is characterized by reduced body skeletal muscle mass, decreased muscle strength, and declined function in physical activity [2]. Sarcopenia affects 10–16% of older people in the world [3]. There is a 5.5–25.7% prevalence of sarcopenia that has been reported by AWGS (Asian Working Group for Sarcopenia) in 2019 [4]. In Hong Kong, 9.4% of elderly people confront sarcopenia [5]. Sarcopenia can cause a decline in physical function in older people and increase the risk of falls and fractures [6]. Sarcopenia is mainly caused by a lack of long-term resistance training and protein intake [7,8]. Not only will it affect the quality of life of older people, but it may also affect the lifespan of the elderly [9]. Therefore, it is necessary to screen and prevent sarcopenia in advance. The European Working Group on Sarcopenia in Older People (EWGSOP2) mentioned in the 2018 consensus that ultrasound imaging, as a non-invasive examination technology, plays a vital role in screening and diagnosing sarcopenia with the advantages of being low-cost, portable, and radiation-free [10]. Ultrasound imaging shows muscle quality by echogenicity and is able to evaluate muscle thickness, cross-sectional area, muscle fiber length, and pennation angle, gastrocnemius muscle, making ultrasound an efficient and effective approach for sarcopenia evaluation [10,11]. Ultrasound technology is used in a variety of medical and health fields, including sports medicine, where it can help monitor serological creatine kinase levels. It can also aid in understanding the relationship between body composition and muscle size, shape, and quality, allowing for a more reliable use of ultrasound technology for muscle health screening [12,13].

The rectus femoris (RF) muscle is one of the most commonly involved anatomical muscles for sarcopenia assessment [14]. It is located in the front of the thigh and is shaped like a fusiform [15]. Together with the surrounding vastus lateralis (VL), vastus intermedius (VI), and vastus medialis (VM), it forms the quadriceps muscle [15,16]. Four muscles merge to create a common tendon, which performs a significant role in leg extension and flexion [15]. RF exerts significant influences on daily activities. For example, the support of the rectus femoris is required in actions such as sitting, standing, walking, jumping, running, climbing stairs, squatting, standing, etc. [17,18]. There are studies to detect RF muscles using computed tomography (CT) and magnetic resonance imaging (MRI) screening [19,20], especially using deep learning methods for RF muscle segmentation using CT and MRI [21,22]. However, there are risks of radiation for routine CT examinations, and it is expensive and inconvenient for people with declining physical abilities to undertake MRI testing on a routine basis as well [23,24]. By contrast, ultrasound has unique advantages over routine assessment of the RF muscle for sarcopenia. Firstly, unlike CT, ultrasound is free from the risk of radiation [23,24]. It is also inexpensive for patients and cost-effective for clinics compared to MRI [23,24]. Secondly, ultrasound is portable and especially convenient for severe sarcopenic patients with disabilities [25,26]. Thirdly, ultrasound is a real-time-based modality and can generate results instantly [27]. These advantages make ultrasound an ideal approach for measuring sarcopenia on a routine basis compared to CT and MRI.

Ultrasound has unique advantages; however, during image segmentation using the RF muscle from ultrasound images, the limitation is that the operator dependency could make interpreting and understanding the RF muscle subjectively, arising from the inter-rater variabilities in manually segmenting the RF muscle for downstream analysis. It is very time-consuming, especially when the doctors are busy; it takes longer for doctors to detect RF muscles without the help of AI. This issue arises naturally during the hand labeling procedure. In light of this, it is crucial to incorporate AI-assisted methods for the demand of RF automatic segmentation, paving the way towards long-term development of full-automated unbiased ultrasound-based sarcopenia assessment.

In the contemporary era of AI, the U-Net deep neural structure and nnU-Net, an improved U-Net structure, have risen to fame swiftly for their classic architecture and high-accuracy training on small sample sizes [28,29]. AutoSAM (Automated Segment Anything), on the other hand, is a newly rise, cutting-edge image segmentation model in recent years. AutoSAM allows automatic promptable segmentation and has been improved based on the Segment Anything (SAM) model [30]. It is efficient in image segmentation, and when providing a given image and marking out the aimed region, SAM can learn the region of interest within the marked area. The mark is regarded as the “prompt” [31,32]. There is no need for clinical operators to mark the prompt by themselves. The promptable segmentation of AutoSAM is able to generate segmentation masks that offer more flexibility in prompts and allow for rapid mask computation and awareness of ambiguity with high accuracy and time efficiency [33].

The existing relevant studies in the literature on ultrasound-based RF muscle segmentation present the following limitations: Firstly, there is a severe lack of relevant studies on AI-empowered automated RF segmentation on ultrasound images. There are very few relevant studies employed classical U-Net models, while the more advanced networks debuted in recent years have not been investigated [34,35]; secondly, one relevant study showed that although the segmentation results were reported to be as high as 0.95 in DICE score, it may not represent the authentic clinical performance of their developed segmentation models as the predicted segments were modified and smoothened before generating the segmentation results; thirdly, the sample size in relevant studies was small and included various age groups instead of specifically designed for older people [36,37,38]. For example, in the study of Ritsche et al., they used images of 1772 from 153 participants with an average age of 38.2 years old to carry out the RF muscle segmentation [38]. In another study by Ritsche et al., they used 60 adolescent and adult soccer players with an average age of 17.8 years old to study the vastus lateralis and RF assessment [37]. In the study of Katakis et al., they acquired 210 subjects, which consisted of 76 elderly people with an average age of 77.3 ± 6.4. They formed a dataset of 2005 ultrasound images containing RF muscle and other muscles [36]. In these studies, younger people’s muscles are much stronger than the elderly. Thus, younger people’s muscle texture and thickness differ from those of older ones with sarcopenia [23,26]. It would be more accurate to train using only the older people’s muscles for their RF muscle segmentation training. Because of the above, in this study, we aim to provide a reference for elderly people’s RF segmentation using cutting-edge AutoSAM models, classic nnU-Net, and U-Net models.

We aimed to benchmark three state-of-the-art networks (U-Net, nnU-Net, and AutoSAM) for RF segmentation in a prospective cohort of older adults in Hong Kong. Of note is that there are no previous studies on advanced promptable networks such as AutoSAM on this topic, which would be more user-friendly from user perspectives when it comes to real-world clinical practice. We evaluated the performance of the developed networks in a broad spectrum of evaluating metrics, including DICE, Intersection over Union (IoU), Hausdorff Distance (HD), Accuracy, Precision, and Recall. The results of this study would provide the community with insights regarding the performance of the state-of-the-art segmentation models for RF muscle, thus offering an alternative solution as an automated, accurate, and objective segmentation method for sarcopenia assessments in the long run.

## 2. Materials and Methods

**Figure 1:**
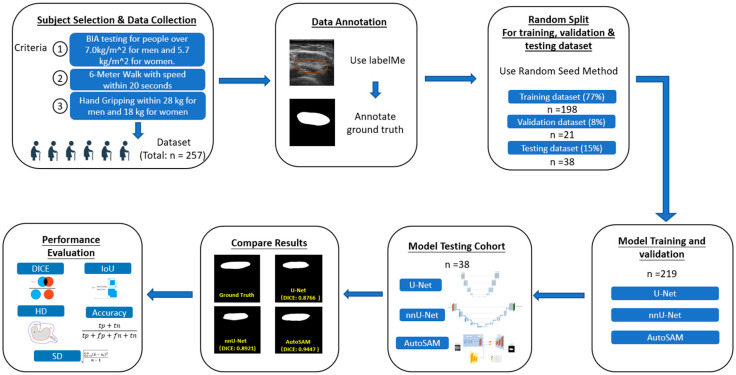
The segmentation process workflow using U-Net, nnU-Net, and AutoSAM. After collecting and annotating data, we used the random seed method for data randomization. After validation, the data were tested with 38 images through all three models and evaluated using a DICE score, IOU, HD, Accuracy, Precision, and Recall.

The whole working schema is shown in Figure 1, which shows the process of the experiment, including subject selection and data collection process, data annotation, random split for training, validation and testing dataset, model training and validation, model testing cohort, compare results, and performance evaluation. Specifically, as mentioned in the Introduction, cross-sectional area is an important and common clinical characteristic of muscle quality. Thus, Bland–Altman analysis and regression analysis are conducted for cross-sectional area measurement between the ground truth and all three models to evaluate the generated results.

### 2.1. Data Acquisition

In this study, we acquired 257 subjects from March 2023 to June 2024 in Hong Kong District Elderly Community Centers to undertake sarcopenia ultrasound screening. The participants were people who were over 65 years old, with an average age of 73.5 ± 6.4 years old. Ethical approval of this study was acquired in July 2022 from the Human Subject Ethics Sub-committee (HSESC) of Hong Kong Polytechnic University (HSEARS20220704004-01). Within these subjects, 183 subjects were diagnosed as sarcopenic according to the protocol provided by AWGS [4]. The criteria for sarcopenia screening are (1) bioelectrical impedance analysis (BIA) testing (BIA InBody S10) for people over 7.0 kg/m^2^ for men and 5.7 kg/m^2^ for women [4,39]; (2) 6-Meter walk with speed within 20 s; and (3) hand gripping within 28 kg for men and 18 kg for women. Subjects who met one of the three criteria would be considered to have sarcopenia.

Subjects experienced ultrasonic evaluation using muscle assessment software (Version 2.0, developed by the Biomedical Engineering Department (BME), The Hong Kong Polytechnic University). The exam was conducted using a linear array transducer of 7.5/10 MHz and 128 elements, which measured the RF at a depth of 40 mm. The evaluation protocols were the same for all subjects.

**Figure 2:**
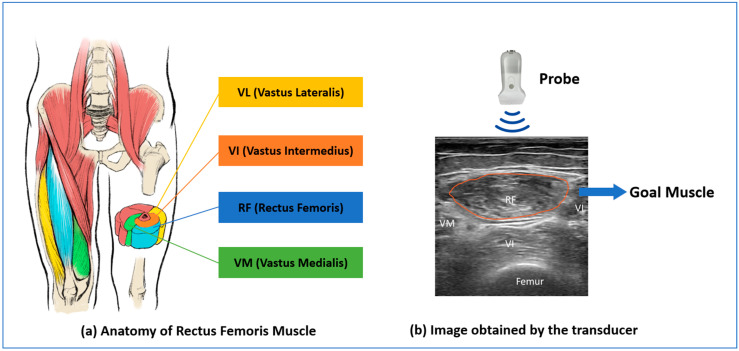
Rectus femoris muscle (**a**) Anatomy of Rectus Femoris Muscle; (**b**) Image obtained by the transducer.

During the ultrasound examination, subjects were required to sit on a chair with feet drawn to the ground. The measurement was undertaken by two nurse practitioners, instructed by a professional medical doctor with ten years of experience. The thigh is paralleled to the floor to show the thigh muscle completely. For subjects, the hips were sitting on the chair, and the thigh muscle was not placed on the chair in order to keep the muscle draping naturally. The scan starts from the knee and moves 10–15 cm to find the rectus femoris muscle, where the probe is perpendicular to the thigh skin surface. A considerable amount of ultrasound gel (Aquasonic Ultrasound Gel, Parker Laboratories, Fairfield, NJ, USA) was applied to the skin under the probe to perceive the utmost ultrasound image. The images are obtained under a gray scale with a size of 384 pixels ∗ 400 pixels. In the image, the muscle around the RF would also be included in the ultrasound image, i.e., vastus intermedius (VI), vastus medialis (VM), and vastus lateralis (VL), as shown in Figure 2.

### 2.2. Database Annotation

**Figure 3:**
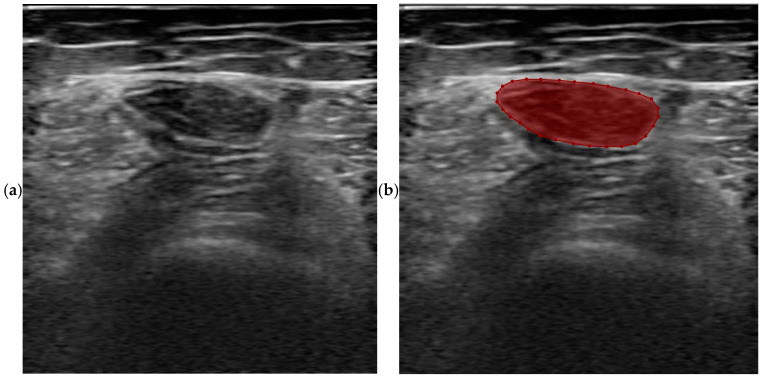
Annotation for rectus femoris muscle (highlighted in red) ((**a**) left: original raw image; (**b**) right: ground truth of annotation of rectus femoris muscle).

Software LabelMe (Version 5.5.0) [40] was used to annotate the cross-sectional area of the rectus femoris muscle under the supervision of a professional ultrasound doctor with ten years of experience. Each image was labeled manually as the ground truth. As shown in the red highlight in Figure 3, (a) on the left is the original image, and the mask on the right is the ground truth labeled. In the annotation process, the physician first started from the upper layer of the rectus femoris muscle to the border between the RF and subcutaneous fat; then, we marked the fascia from the surrounding fascia between RF and VM, VI, and VL. Finally, we turned the saved LabelMe file into a JSON file format and transferred JSON files into masks for model segmentation. All original images and masks share the size of 384 ∗ 400.

### 2.3. Neural Networks of Deep Learning and Segmentations

Clinically, we selected the three structures of U-Net, nnU-Net, and AutoSAM to perform a comprehensive benchmarking study. Neural Networks play a pivotal role in image segmentation. Among all kinds of neural networks, U-Net [28] and nnU-Net [29] are two models that are popular in medical segmentation [34]. AutoSAM, the automatic promptable model, is one of the most pioneering models nowadays [30] and was also selected. These models were chosen from numerous state-of-the-art deep-learning models used for muscle segmentation.

We first selected U-Net for its classical and traditional status in a segmentation model and its effective performance in supervising segmentation tasks [28,41]. Furthermore, the nnU-Net structure was chosen for its enhancements over the traditional U-Net model [29,41]. The nnU-Net model is an adaptive, unified, extensible training framework, making it a promising choice for various medical image segmentation tasks [41]. Its superior performance across datasets and potential for pre-trained models further solidify its position as an effective tool for medical image analysis [41]. Most importantly, our selection of AutoSAM is superior to the SAM model for its automated prompting process, and SAM has been the latest segmentation structured for medical usage with its unique promptable network [30]. In this case, we aimed to fill the gap by using the classic and the most cutting-edge architecture for RF muscle segmentation to assess sarcopenia.

The random seed method [42] was adopted for model training and testing. The training set and validation set consisted of 85% of the whole dataset; the training set contains 219 subjects (198 subjects for training and 21 subjects for validation, which accounts for 77% and 8% of the whole dataset), and the testing set consisted of 38 subjects (15% of the whole dataset). Each image represented a subject, and there was no overlap in the subjects among the training, validation, and testing datasets. Figure 3 shows the segmentation process workflow using U-Net, nnU-Net, and AutoSAM. After collecting and annotating data, we used the random seed method for data randomization. After validation, the data were tested with 38 images through all three models and evaluated using a DICE score, IOU, HD, Accuracy, Precision, and Recall.

#### 2.3.1. U-Net

**Figure 4:**
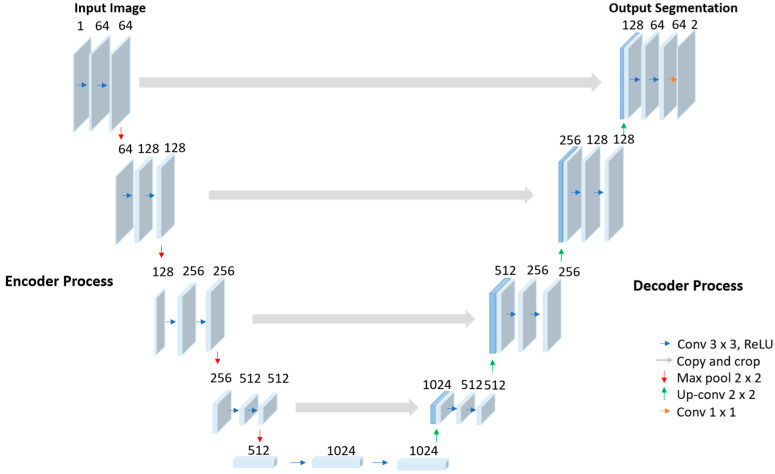
Model structure of U-Net [28].

U-Net is a popular and classic image segmentation architecture widely used in the medical field [43,44]. The architecture comprises a contracting pathway for context acquisition and is able to learn from a small training sample at a fast speed with high accuracy [28]. Figure 4 shows the architectural structure of U-Net with down-sampling of the encoder and up-sampling of the decoder for extracting and integrating features. Features are transmitted through the encoder and decoder utilizing successive convolutional layers and a max pooling layer, along with extracted intermediate features. The recovered features are up-sampled by the corresponding decoder, which integrates up-sampling decoders with preserved copies of the encoder features, allowing concatenation with the decoder features through related components. The final layer is capable of generating the output.

The encoder consists of a sequence of successive 3 × 3 convolutional layers. Following each convolutional layer, the rectified linear unit (ReLU) activation function is applied to each feature. A 2 × 2 max pooling process down-samples the features between the stages. The channel is multiplied by two following each down-sampling operation. The decoder consists of several repeated 3 × 3 convolutional layers, with ReLU activation applied to each feature. The decoder upscales the features and implements 2 × 2 convolutional layers, reducing the number of channels by half. The up-sampling technique is employed to restore the spatial resolution of features diminished during the encoder phase. In this RF segmentation experiment, we set the learning rate of 0.00001 with 0.5 in scale for 100 epochs.

#### 2.3.2. nnU-Net

nnU-Net refers to “no-new-Net” and is developed based on the U-Net structure [29]. It is also an automatic segmentation model that contains the process of preprocessing, structural architecture, model training, and post-processing that promotes accuracy in medical imaging segmentation [41]. nnU-Net optimized and generated adaptive processing based on the classic U-Net structure, the 2D and 3D U-Net. nnU-Net is capable of automatically adjusting model structure and training parameters to improve the architecture, preprocessing, and training process with stronger model robustness and higher data quality; for example, nnU-Net uses instance normalization compared to U-Net’s use of batch normalization and updates the ReLUs. nnU-Net also upgraded the training steps involving resampling and regularizing the preprocessing process.

**Figure 5:**
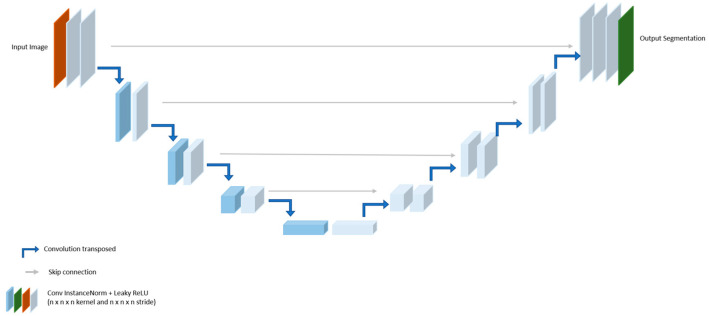
Model structure of nnU-Net with InstanceNorm and LeadkyReLU under Transpose [29].

nnU-Net shares a similar architecture with the U-Net model, as shown in Figure 5; it also starts with an encoder and a decoder with the process of up-sampling and down-sampling. Encoder is used for feature extractions and gradually minimizes the resolution. The decoder is used to recover the resolution for the mask prediction. By improving the network depth, nnU-Net is able to measure the similarity between the ground truth and the predicted masks. In each process, the convolution transposed the features to the next depth. With leaky ReLU, nnU-Net is able to enhance the model training speed and quality since it can deal with the negative value input and avoid the ceasing of pre-activation; thus, it could improve the model’s performance. In the RF segmentation experiment, we used the open-sourced codes for nnU-Net.

#### 2.3.3. AutoSAM

AutoSAM (Segment Anything Model) model is an automatic promptable image segmentation tool with high efficiency that is generated from the SAM model [30]. However, SAM faces challenges when segmenting medical images because it was initially limited to natural images. AutoSAM, on the other hand, generates prompts automatically, which saves time for pretraining processes such as fine-tuning [30]. AutoSAM could help prompt generation using input images, which differs from a “U” structure incorporating an encoder and decoder for up-sampling and down-sampling. AutoSAM could freeze the SAM encoder and perform light-weight fine-tuning. By overloading the prompt encoder, AutoSAM could be improved by proposing a new encoder on the input images that replaced the original prompt encoder [45].

**Figure 6:**
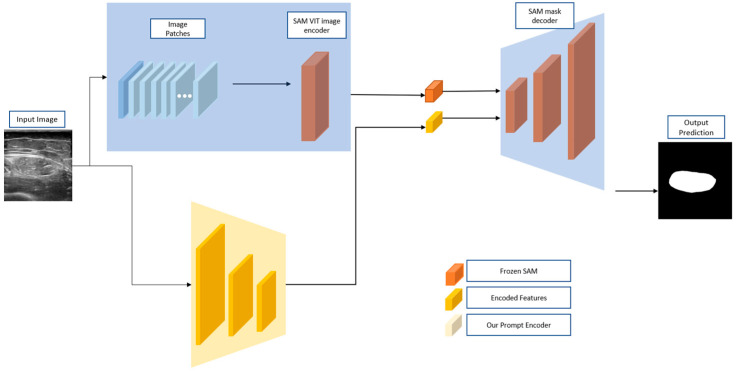
AutoSAM structure of replacing prompt encoder with custom encoder when the image encoder and mask decoder are frozen in SAM [30].

The model first starts with the input images as prompts, and then the image patches are transferred to SAM Vision Transfer (ViT), the image encoder. Then, the ViT could predict the Head using the SAM decoder. Thus, the decoder would generate the output mask and remove the prompt token in the auxiliary embedding so that it is no longer a promptable model. In Figure 6, the encoder is presented with higher accuracy without a fine-tuning process. Also, the method involves training a prompt generator network that generates guidance prompts for SAM based on the input images. In the RF segmentation experiment, we used the open-sourced codes for AutoSAM.

### 2.4. Evaluation Methods and Metrics

The same testing set is used to assess the model segmentation agreement of our three models. Evaluation matrices usually contain the DICE score, IoU, HD, Accuracy, Precision, and Recall [46,47]. The DICE score is one of the most popular evaluation parameters. The following shows the equations of the five evaluation matrices.

#### 2.4.1. DICE

DICE represents the Dice coefficient (i.e., Dice similarity) and is usually used to evaluate the accuracy in image segmentation tasks. DICE measures the overlap between the segmentation mask generated by the model and the ground truth. The calculation equation is shown in Equation (1). A represents the pixel set of the segmentation result generated by the model, and B represents the actual label pixel set. The value range of the DICE coefficient is set between 0 to 1.

(1) $$dice=\frac{2|A⋂B|}{\left|A\right|+|B|}$$

Equation (1). Dice Score Calculation Equation.

#### 2.4.2. IoU

IoU represents the “intersection over union”, and it measures the overlap between the results generated by object detection or image segmentation algorithms and the actual annotations. The value range of IoU is between 0 and 1, where 1 means complete overlap and 0 means no overlap.

(2) $$IoU=\frac{\left|A\cap B\right|}{\left|A\cup B\right|}$$

Equation (2). IoU Value Calculation Equation.

#### 2.4.3. HD

HD refers to Hausdorff Distance, a distance metric that quantifies the similarity between two shapes [48]. It is generally used to measure the similarity between the segmentation results generated by the algorithm and the actual label. A smaller HD value indicates a higher similarity between the two shapes; the segmentation result is closer to the actual label. The calculation equation is shown in Equation (3).

H(A,B) = max(H(A,B),H(B,A))(3)

Equation (3). Hausdorff Distance.

#### 2.4.4. Accuracy, Precision, and Recall

Accuracy is calculated by the pixels of the image. In the confusion matrix, the predicted case is either positive (P) or negative (N), and the actual situation case is either true (T) or false (F). When the predicted case is positive, and the real situation case is true, we mark this scenario as “true positive”, which is tp. Correspondingly, when the predicted case is negative, and the real situation is true, the “true negative” scenario is regarded as tn. The other two types of combination, “false positive” (fp) and “false negative” (fn), are regarded as Type I errors and Type II errors. The calculation of the accuracy is referred to as the sum of correct prediction, namely the sum of tp and tn divided by all scenarios, which is the sum of tp, fp, fn, and tn. The calculation equation of Accuracy is the sum of tp and tn and divided by the sum of tp, tn, fp and fn. Precision is calculated with tp divided by the sum of tp and fp. Recall measures tp divided by the sum of tp and fn.

#### 2.4.5. Standard Deviation

Standard Deviation (SD) measures the extent to which the data are scattered from the mean value. A larger SD indicates a wider data spread; a smaller SD indicates the data points are closer to the mean. The calculation process first calculates the average squares of the distances that the data deviate from the mean, then calculates the square root to measure the degree of dispersion.

## 3. Results

A total of 257 subjects were measured for their RF muscle using segmentation tools of U-Net, nnU-Net, and AutoSAM. After conducting our experiment, our study presented the results of the DICE, IoU, HD measurements, accuracy, precision, and recall, as shown below in Table 1, with SD representing standard deviation. Our results are shown by the comparison of the testing set to the training set. Quantitively, AutoSAM demonstrated superior performance compared to U-Net and nnU-Net, achieving the highest metrics in all evaluation matrices: DICE score is 0.9205 (SD = 0.0449), IoU is 0.8557 (SD = 0.0720), HD is 1.7213 (SD = 1.1670), Accuracy is 0. 9826 (SD = 0.0137), Precision is 0.9897 (SD = 0.0109), and Recall is 0.9911 (SD = 0.0141). In the context of U-Net, the DICE score is 0.8178 (SD = 0.1010), IoU is 0.7035 (SD = 0.1371), HD is 4.2724 (SD = 2.6113), Accuracy is 0.9637 (SD = 0.0175), Precision is 0.9828 (SD = 0.0189), and Recall is 0.9759 (SD = 0.0221). In the context of nnU-Net, the DICE score is 0.8910 (SD = 0.0610), IoU is 0.8084 (SD = 0.0924), HD is 3.2074 (SD = 2.7506), Accuracy is 0.9789 (SD = 0.0145), Precision is 0.9881 (SD = 0.0143), and Recall is 0.9884 (SD = 0.0142). We evaluated model stability by SD, which was calculated based on the DICE value of each image in the test set. It showed that AutoSAM has the smallest SD among all the evaluation indexes. Thus, AutoSAM has the best stability among all three models as well.

**Figure 7:**
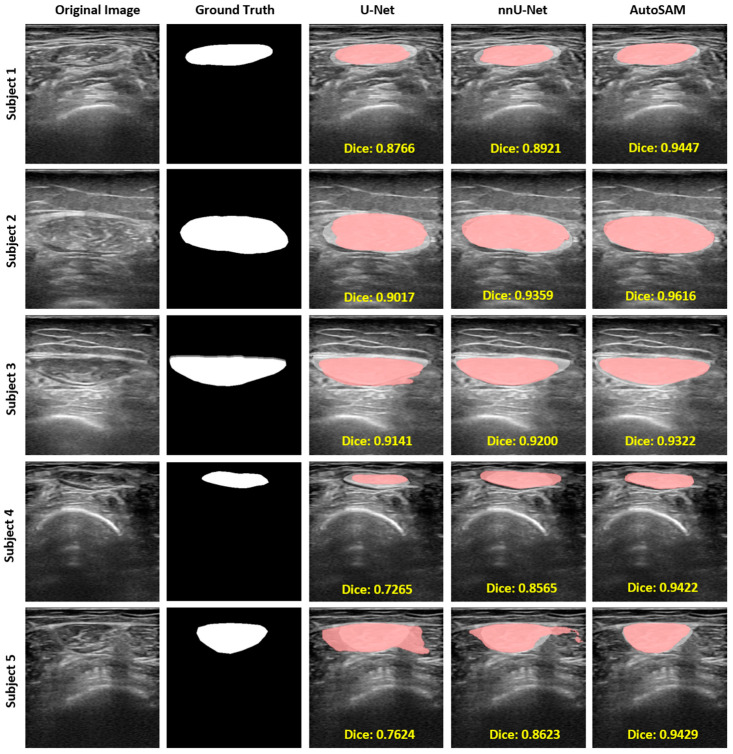
Examples of graphical examples using U-Net, nnU-Net, and AutoSAM.

**Figure 8:**
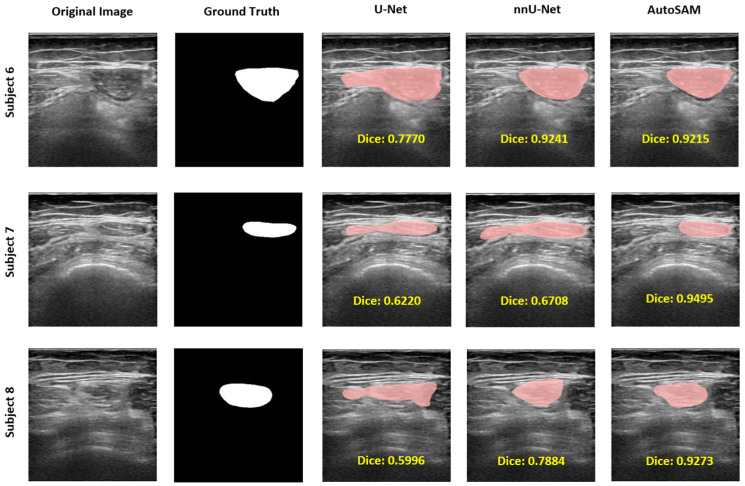
Examples of clinical examples showing segmentation limitations.

The results were illustrated qualitatively. Figure 7 presents examples illustrating the outcomes produced by all three models and showcases the detailed results of the masks generated by the three models. The white region represents the ground truth annotation, and the red region represents the mask generated by each model. Remarkably, AutoSAM consistently yielded the best result compared to U-Net and nnU-Net, as shown in Figure 7. Compared to U-Net and nnU-Net, AutoSAM is able to generate a precise border as ground truth. It can be seen that the mask generated by U-Net has redundant boundaries, some boundaries cannot be well identified, and some boundaries are even missing. U-Net detects RF muscle roughly. For example, in subjects 1, 2, and 4, we could see the U-Net generated a rough contour of the RF muscle. The red region for U-Net and nnU-Net shows the non-overlap region with the white region, which indicates that the predicted mask does not fully align with the ground truth. In contrast, the white region in AutoSAM could align well with the predicted red region, as shown in examples 3 and 5. In sum, the result generated by the U-Net does not fully align with the ground truth. nnU-Net has made some progress on the contour and border, but the abovementioned problems still exist, as in example 5, where the right part is obviously inaccurately detected. On the contrary, AutoSAM is much more accurate in identifying boundaries than U-Net and nnU-Net, with no excessive or missing boundaries to be identified. Thus, AutoSAM recognizes boundary ranges more accurately and smoothly. However, there are some limitations in segmentations. As shown for subjects 6, 7, and 8 in Figure 8, U-Net did not perform well on segmentation for all three examples. For subject 7, nnU-Net did not segment very clearly as well. For subject 8, AutoSAM did not generate a clear and smooth border compared to the ground truth.

**Figure 9:**
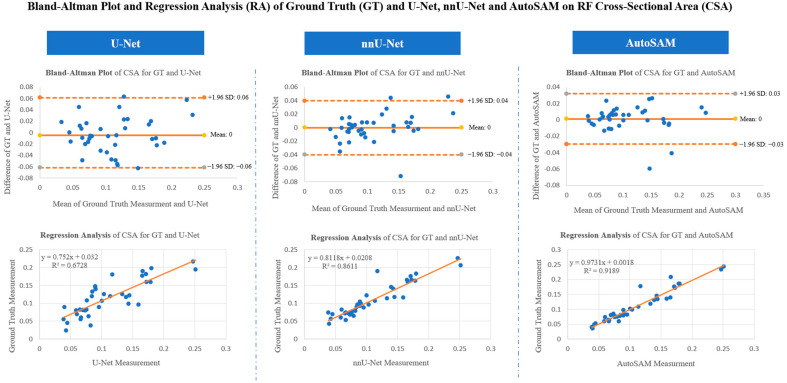
Bland–Altman analysis and regression analysis comparing ground truth vs. U-Net, nnU-Net and AutoSAM results.

Figure 9 shows the Bland–Altman plot and regression analysis of the ground truth (annotated manually) and model-generated results of U-Net, nnU-Net, and AutoSAM. We can see that the differences between the ground truth and models-generated results are not remarkable, as they both fall within the range of 1.96 standard deviations, with only a few outliers falling outside of the standard deviation borders.

The correlation between ground truth and AutoSAM is strong (r^2^ = 0.9189), as well for the nnU-Net (r^2^ = 0.8611). The correlation is moderate for the ground truth and U-Net (r^2^ = 0.6728). From the Bland–Altman and Regression analysis, we could see that AutoSAM outperformed nnU-Net and U-Net.

## 4. Discussion

Sarcopenia is now receiving growing attention across the globe, particularly in view of the rapidly expanding aging population worldwide. RF, as an important muscle for evaluating sarcopenia, has been explored by various modalities in recent years. RF segmentation plays an important role in sarcopenia assessment. A clear and accurate detection of sarcopenia enables researchers to understand and assess the severity of sarcopenia more efficiently. The role of imaging in diagnosing sarcopenia is frequently utilized in older outpatients and inpatients, and ultrasound is especially popular in all imaging techniques [49]. We used ultrasound rather than MRI or CT to measure RF muscle for the following reasons: it is non-invasive, radiation-free, economical-friendly, and portable. With the help of ultrasound, we are able to detect RF muscle and its surrounding muscles of VI, VM, and VL. Interpretation of the RF muscle segmentation using ultrasound could vary tremendously due to subjective understanding during the annotation. Thus, a fully automatic ultrasound-based segmentation algorithm is helpful for the study of sarcopenia. Incorporating AI into RF segmentation allows us to segment the muscle more efficiently and effectively. Our goal was to evaluate the performance of deep learning models on RF segmentation in our local cohort of older people with sarcopenia. We utilized U-Net, nnU-Net, and AutoSAM models and found that AutoSAM yielded the most accurate results quantitively and qualitatively that quantitively AutoSAM yields the highest Dice score; and qualitatively, the Bland–Altman shows that AutoSAM has the narrowest range of the points scattering that fits the range of agreements to the ground truth; and AutoSAM yields the highest correlation r^2^ among all three models. The qualitative results yield consistent results with the Dice score and other evaluation metrics, providing concrete evidence showing AutoSAM outperformed the other two models.

In our study, we adopted two types of segmentation models for RF muscle: U-Net structured model (U-Net, nnU-Net) and prompt-based model (AutoSAM). The quantitative and qualitative results of the evaluation are shown in Table 1 and Figure 7. The first two models are based on the U-Net structure with an encoder and decoder from down-sampling to up-sampling. In contrast, the AutoSAM model is based on the promptable model with the enhancement of a no-prompt structure. U-Net and nnU-Net involve a decoder and an encoder, whereas AutoSAM is developed based on the SAM, and it is able to generate results with automatically initiated prompts. We selected U-Net and nnU-Net for their unique status in the medical imaging segmentation field. U-Net is a deep learning network with a U-shaped architecture. It is a classic convolutional neural network and one of the most used image segmentation tools. nnU-Net is derived from the classic U-Net structure with improved performance in lesion detection [50]. U-Net-based structure has many advantages in medical segmentation. First, the features in medical images are important and could be hard to capture in ultrasound images due to the echogenicity. The U-shaped skip connection structure is able to splice features. Second, medical data usually has a small number of hundreds of images; in our case, we have 257 images, which makes it easy to be overfitted. This convolutional neural network is able to deal with fewer medical images. However, U-Net and nnU-Net could still have problems detecting unclear borders and recognizing irrelevant information. SAM-based architecture has been trained with more than one billion masks and is able to use a promptable structure to learn the new image by offering a prompt. AutoSAM is able to generate prompts automatically, thus making AutoSAM a more efficient segmentation tool 30]. Although SAM-based architecture marks the epoch of using the prompt-driven paradigm for image segmentation to enhance efficiency and accuracy, limitations still exist in the application’s viability and stability. For images with irregular shapes, weak boundaries, small size, or low contrast, SAM is not able to yield satisfactory performance consistently for every experiment [51].

Previously, research groups that investigated RF muscle segmentation encountered certain problems. Marzola et al.’s study evaluated tibialis anterior, gastrocnemius medialis, and biceps brachii using U-Net, U-Net++, and Attention U-Net. Finally, they reached the DICE score of 0.88–0.89. However, the models were limited to the U-Net-related structures [52]. Yet, another study also focused on the convolutional network to evaluate quadriceps from ultrasound and reached the DICE score of 0.90 for 73 subjects. However, the dataset is relatively small [53]. Our study considered it important to include only older people for the exam since muscle performs differently and deteriorates as people age. The fat infiltration within muscles could affect ultrasound images [54,55,56]. Fat infiltration would also affect ultrasound imaging, and fat infiltration increases skeletal muscle echo intensity and affects ultrasound images, making the muscle whiter on the ultrasound image [57]. Thus, the muscles of subjects of different age groups have different muscle characteristics [58,59,60]. In this case, our dataset is more convincible and valuable in elderly sarcopenia RF muscle detection evaluation.

Accurate data selection helps us better understand clinical research on sarcopenia in the real world. Our dataset selection process is stricter and is subject-based instead of image-based, which is more scientifically correct than one subject providing one image. And that image goes to either the training, validation, or testing sets. In related research, although the images were not the same within the training and testing sets, the images could be from the same subject [34,36]. From our viewpoint, each subject should only provide one single image because when selecting more than one image from a subject, the images could carry similar information. Thus, the characters of the same subject would be brought into the training and testing dataset, i.e., the characteristics and features of one subject could affect the result in the testing set, which might generate a higher accuracy in the dice score. In this case, the high dice score could not fully show the real accuracy in the testing set. It could overestimate the accuracy since features are extracted similarly in both the training and testing datasets. Therefore, when choosing the subject-based dataset, we followed the protocol that each subject provided one image, and there was no overlap between the training and testing datasets.

Moreover, we looked beyond merely the accuracy result with a high dice score; instead, we focused more on clinical values within the dataset. Clinically, our data processing is more authentic than raw data without post-processing, which could help us understand the real cases of elderly muscle segmentation. Post-processing could help generate a high DICE score. However, these realistic results could impede doctors and healthcare providers from obtaining the real picture of the muscle. One relevant study in the literature by Katakis et al. [36] post-processed the result with refinement to discover the cross-sectional area, and the results of all models were 0.95 in the DICE score. However, they used post-processing for raw results due to the possible artifacts and noise. In our study, our goal is to discover the segmentation performance in each model, and we need the raw results to reflect the performance of each model, so we did not include post-processing. From the results, AutoSAM differs from traditional convolutional neural networks because it generates higher accuracy and is less likely to generate artifacts and noise. In this case, post-processing is unnecessary for AutoSAM. 

Despite all the inspiring results, the study also presents several limitations. Firstly, our study is limited to data collection and contains only one cohort. We are eager to incorporate different cohorts, such as clinical and in-home case cohorts, in addition to older people’s home cohorts. Secondly, we did not make classifications within genders, age groups, and diseases. As the model develops, more structures and networks begin to emerge, and in our study, the model comparison is still limited. Besides segmentation, integrating classification is also important. In levels of the algorithm, it is necessary to involve classifications such as diffusion networks [61], dual-channel CNXV2-DANet [62], and automated image classification [63] model to enhance our model variety on sarcopenia severity classification. In the future, we are eager to see a more complex and novel structure for comparison.

## 5. Conclusions

The AutoSAM model yielded the best RF segmentation results qualitatively and quantitatively compared to the U-Net and nnU-Net models. The results of this study evaluated the segmentation models from classic, popular U-Net-based structure (U-Net and nnU-Net) to the cutting-edged promptable SAM model (AutoSAM). Despite the abovementioned limitations, the study proved that fully automated ultrasound-based segmentation can detect RF muscles accurately. The outcomes serve as valuable references for future studies and are the cornerstone of the study of RF muscle for sarcopenia from clinical settings to community settings, even to in-home care. We selected a dataset that only contained the elderly. We also used a subject-based dataset selection basis that subjects in the testing dataset do not overlap with subjects in the training dataset. The results of this study would provide inspiring insights and could encourage researchers within the relevant fields to step further on ultrasound usage in sarcopenia assessment that incorporates AI-assisted methods, thus could be beneficial for clinical and community healthcare providers to prevent, assess, and treat sarcopenia in today’s fast-paced, advanced society.

## Figures and Tables

**Table 1:** Results of segmentation agreement (with evaluation matrix of DICE, IoU, and HD) and stability (with standard deviation (SD)) for U-Net, nnU-Net, and AutoSAM model. The top-ranked results are marked in bold.

Model	DICE	IoU	HD	Accuracy	Precision	Recall
U-Net	0.8178 (SD = 0.1010)	0.7035 (SD = 0.1371)	4.2724 (SD = 2.6113)	0.9637 (SD = 0.0175)	0.9828 (SD = 0.0189)	0.9759 (SD = 0.0221)
nnU-Net	0.8910 (SD = 0.0610)	0.8084 (SD = 0.0924)	3.2074 (SD = 2.7506)	0.9789 (SD = 0.0145)	0.9881 (SD = 0.0143)	0.9884 (SD = 0.0142)
**Auto-SAM**	**0.9205 (SD = 0.0449)**	**0.8557 (SD = 0.0720)**	**1.7213 (SD = 1.1670)**	**0.9826 (SD = 0.0137)**	**0.9897** **(SD = 0.0109)**	**0.9911** **(SD = 0.0141)**

## Data Availability

Dataset available on request from the authors.
